# Supplementary material for: Acceptance and compliance with micronutrient powder (MNP) among children aged 6–23 months in northern Nigeria
Source: PLOS Glob Public Health. 2022 Oct 17;2(10):e0000961. doi: 10.1371/journal.pgph.0000961 (PMC10022258; doi:10.1371/journal.pgph.0000961)
Supplement: S1 Dataset — (ZIP) [file pgph.0000961.s008.zip › S8_Dataset/Direct Observations.zip!/Direct Observations/220605_A_GA_AD_1_DO.pdf]

1.1.1 Instrument F –Direct Observation Form

**<<Instrument F Direct Observation Form>>**
**PI Name:** PragyaMathema,Stephen Kodish<br>**Study Title:** Formative research to develop an infant and young child feeding (IYCF) intervention with micronutrient powder (MNP) for children aged 6 – 23 months<br>**PI Version/Date:** Version1.0/January 24, 2015

Date: _June 22, 2015_____________Location: _ Dadiri community,GANYE, LGA_________

Data Collector: Aisha Aliyu Dahiru_______________Participant ID: __small hut with low verandah_

Main goals of this direct observation are to answer the following questions:
1. In this household, what would be the best way(s) to give nutritional supplements to young children? Why?<br><br>2. In this household, describe the food sharing that you observe throughout the meal. Are there any predictable patterns?<br><br>3. In this household, who is favored and how is this favoritism expressed during meal preparation and eating?

(TEAR OFF AT PERFORATED DOTTED LINE BELOW)

PARTICIPANT ID: small hut with low verandah__

Household Composition Table (Fill out as you do data collection)

INITIALS	SEX (M/F)	AGE (estimated)	Relationship to household	Description
M	F	30YRS	Mother	Wearing a red wrapper with a head tie.
BB	M	18 MONTHS	BABY BOY	Dark, wearing pants without shirt because of wound on his chest.
BC	M	2 YEARS	Sister's son (boy)	Wearing blue checked shirt with brown trouser.
OB	M	8 YEARS	Older son	Wearing blue shirt and black trouser
OS	M	11YEARS	Older son	Wearing blue shirt and black trouser
N	F	17 YEARS	Neighbor	Wearing blue dress

PARTICIPANT ID: _small hut wit low verandah________

TIME	ACTOR INITIALS	RECIPIENT INITIALS	WHAT HAPPENED	FOOD	Reactivity
9.00	M	BB, BC	Mother playing with children		
9:05	M	BB,BC	Mother playing with children		
9:10	M	BB,BC	Mother playing with children		
9:15	BB		Baby boy playing with cousin (sister's son)		
9:20	BC		Sister's son playing with baby boy		
9:25	M		Sitting on a bench with the children		
9:30	M	BB,BC	Mother chatting with the children		
9:35	M	BB,BC	Mother chatting with the children		
9:40	M	BB,BC	Mother chatting with the children		
9:45	M	BB,BC	Mother chatting with the children		
9:50	M		Resting under the veranda		

TIME	ACTOR INITIALS	RECIPIENT INITIALS	WHAT HAPPENED	FOOD	Reactivity
9:55	BB		Baby playing near the mother		
10:00	BB		Baby is playing		
10:05	BB		Baby is playing on the bench		
10:10	BB		Baby is playing on the bench		
10:15	BB		Baby is playing on the mat		
10:20	M	BB	Mother breastfeeding baby		
10:25	M	BB	Mother breastfeeding baby		
10:30	M	BB	Mother breastfeeding baby		
10:35	M	BB	Mother breastfeeding baby		
10:40	BB		Baby playing on the mat		
10:45	BB		Baby crying		
10:50	M	BB	Mother carrying baby on her laps		
10:55	M	BB	Mother consoling baby		
11:00	M	BB	Mother carrying baby on her laps		
11:05	M		Mother goes into the room to pick something (flask)		
11:10	M	BB	Mother breastfeeding the baby on the bench		
11:15	M	BB	Mother breastfeeding the baby on the bench		
11:20	M		Mother pouring kunu from the flask		
11:21	M		Pouring a portion of kunu from a big cup into a smaller cup		
11:23	M		Adding MNP into a smaller cup	Kunu gyada	

TIME	ACTOR INITIALS	RECIPIENT INITIALS	WHAT HAPPENED	FOOD	Reactivity
11:24	M		Mixing MNP into kunu	Kunu gyada	
11:24	M		Mixing MNP into kunu		
11:25	M	BB	Mother feeding the baby with kunu using spoon (portion mixed with MNP)	Kunu gyada	
11:26	M	BB	Mother feeding baby kunu mixed with MNP	Kunu gyada	
11:27	M	BB	Mother feeding baby kunu mixed with MNP	Kunu gyada	
11:28	M	BB	Mother feeding baby kunu mixed with MNP	Kunu gyada	
11:29	M	BB	Mother feeding baby with kunu without MNP	Kunu gyada	
11:30	M	BB	Mother feeding baby with kunu	Kunu gyada	
11:35	M	BB	Washing the baby's hands and mouth		
11:40	M	BB	Carrying baby on her back		
11:50	M		Picking plates around the house into a large tray		
11:55	M		Mother taking water from the clay pot into a large container		
12:00	M		Mother taking utensils and water to the back of the kitchen		
12:05	M	BB	Carrying the baby on her back		
12:10	M		Rinsing the utensils		
12:15	M		Stepping out of the house		
12:20	M		Buying detergent from neighbour's		
12:20	M	BB	Mother carrying baby on her back		
12:25	BB		Crying		
12:30	M	BB	Mother trying to console		

TIME	ACTOR INITIALS	RECIPIENT INITIALS	WHAT HAPPENED	FOOD	Reactivity
12:35	M	BB	Brings baby down from back to sit on her lap		
12:40	M	BB	Mother breastfeeding baby		
12:41	M	BB	Mother breastfeeding baby		
12:42	M	BB	Mother breastfeeding baby		
12:43	M	BB	Mother breastfeeding baby		
12:44	M	BB	Mother breastfeeding baby		
12:45	M	BB	Mother breastfeeding baby		
12:50	M	BB	Mother breastfeeding baby		
12:50	BB		Sleeping on mother's lap		
12:55	BB		Sleeping on mother's lap		
01:00	BB		Sleeping on mother's lap		
01:05	M	BB	Lying down baby on the mat under the veranda		
01:05	M		Lying down beside the baby on the mat		
01:10	BC		Lying down beside the mother		
01:15	BB		Baby sleeping		
01:20	M		Sitting beside the children on the mat		
01:25	OB		Walks into the house		
01:25	OB	M	Sitting on a stool and greeting the mother		
01:30	OB		Bringing down a bowl		
01:30	OB		Serving tuwo from bowl into a cup	Tuwon masara	
01:35	M	OB	Scolding the older boy for letting		

TIME	ACTOR INITIALS	RECIPIENT INITIALS	WHAT HAPPENED	FOOD	Reactivity
			the food fall		
01:40	OB		Crying because the mother is scolding him		
01:45	OB		Adding water into cup with tuwo		
01:50	OB		Washing hands		
01:55	OB	BC	Gives sister's son portion of mixed tuwo in a small cup	Tuwon masara	
02:00	OB		Eating the mixed tuwo with his hands	Tuwon masara	
02:00	BC		Eating the mixed tuwo with his hand		
02:00	BB		Baby is still sleeping		
02:00	M		Mother sitting beside sleeping baby		
02:05	OB		Eating with his hands		
02:06	OB	BC	Older boy assisting sister's boy to eat		
02:07	OB		Older boy eating		
02:12	M	OB	Chatting with older boy		
02:17	OB		Finished eating tuwon and licking his hands		
02:20	OB		Washing hands		
02:20	OB		Going out of the house		
02:20	BB		Baby still sleeping		
02:20	BC		Finished eating and handover cup to mother		
02:20	M	BC	Washing boy mouth and hands		
02:25	M		Mixing tuwo with water in a large cup	Tuwon masara, water	
02:25	M	BC	Gives sister's son more of mixed	Tuwon	

TIME	ACTOR INITIALS	RECIPIENT INITIALS	WHAT HAPPENED	FOOD	Reactivity
			tuwo	masara	
02:25	BB		Baby still sleeping		
02:25	M		Washing utensils		
02:30	M		Washing utensils		
02:35	M		Washing utensils		
02:40	M		Washing utensils		
02:45	M		Washing utensils		
02:50	M		Washing utensils		
02:55	M		Washing utensils		
03:00	M		Washing utensils		
03:00	BC	M	Handing cup to mother		
03:00	M	BC	Washing baby’s hands and mouth		
03:00	BB		Baby is sleeping		
03:00	BB		Baby is sleeping		
03:05	M		Washing utensils		
03:10	M	BB	Mother checking on sleeping baby		
03:15	OC		Comes into the house carrying a bag pack		
03:15	OB		Comes into the house along with older brother		
03:20	OB		Changing cloth into brown shirt with canvas		
03:20	M		Mother washing the clay pot containing		
03:25	M		Setting fire		
03:30	M		Setting fire		

TIME	ACTOR INITIALS	RECIPIENT INITIALS	WHAT HAPPENED	FOOD	Reactivity
03:35	BB		Wakes up crying		
03:35	OC	MM	Picking up the baby boy and hands him to mother		
03:35	M	BB	Carrying baby on laps and breastfeed him		
03:40	M	BB	Breastfeeding the baby		
03:45	OC		Picks up plastic container and goes out of the house		
03:45	M	BB	Breastfeeding the baby		
03:50	OC		Bringing in water in plastic container and putting into clay pot		
03:50	M	BB	Breastfeeding the baby		
03:55	OC	BB	Older child playing with baby boy		
04:00	OC		Older child carrying the baby boy		
04:00	M		Setting fire		
04:05	M		Pacing pot with water on fire and covers it		
04:10	M		Adding flour into boiling water to make meal	Maize flour, water	
04:15	M		Meal is ready to be served		
04:15	M		Adding vegetable in the another pot on the fire	Beans leaves	
04:15	M		Adding seasoning into pot on fire and mixing it		
04:20	M		Meal is ready to be served	Beans leaves soup	
04:20	OC	BB	Playing with baby		
04:25	M		Sewing meal into a single dish	Maize meal plus beans leaves soup	
04:30	BB		Playing		
04:30	OC	BB	Giving baby boy water to drink	Water	
04:35	M	OC,BB	Mother chatting with older child and baby under the veranda		
04:40	BB		Playing with chicken		

TIME	ACTOR INITIALS	RECIPIENT INITIALS	WHAT HAPPENED	FOOD	Reactivity
04:40	BB		Playing around the house with older son		
04:45	BB		Playing around the house with older son		
04:50	BB		Crying from a fall while running around		
04:55	M	BB	Mother bathing baby		
05:00	M	BB	Mother bathing baby		
05:05	M	BB	Mother rubs oil and ointment on the baby especially on the wounds on his chest		
05:10	M	BB	Mother dressing up baby		
05:15	N	M	Comes in greeting the mother		
05:15	M	N	Mother welcomes neighbor and offers her on a seat		
05:20	M	N	Mother chatting with neighbor and offers her seat		
05:20	OC	BB	Playing with baby boy		
05:25	M		Goes into room and brings out a packet of MNP		
05:25	M		Mother showing neighbor MNP and chatting		
05:30	M	N	Chatting with neighbor on MNP		
05:30	BB		Playing with older son		
05:35	BB		Playing with older son		
05:40			Playing with older son		
05:40	BB		Pulling bag		
05:45	M	BB	Mother breastfeeding baby		
05:50	M	BB	Mother breastfeeding baby		
05:55	M	BB	Mother breastfeeding baby		
06:00	M	BB	Mother carrying baby on her back		
06:05	M	BB	Mother carrying baby on her back		
06:10	M	BB	Mother brings down baby from back		
					R

TIME	ACTOR INITIALS	RECIPIENT INITIALS	WHAT HAPPENED	FOOD	Reactivity

TIME	ACTOR INITIALS	RECIPIENT INITIALS	WHAT HAPPENED	FOOD	Reactivity

PARTICIPANT ID: __ _______

Primary Questions (Complete <u>after</u> you complete data collection):

From what you have seen observing THIS HOUSEHOLD, please answer the following questions in detail:

Question	Write your <u>detailed</u> response here
1. In this household, what would be the best way(s) to give nutritional supplements such as an MNP to young children to ensure compliance?<br><br>Why?	The best way is to train the mother on MNP because the child is fed from a separate bowl. The mother should be made to understand the importance of giving the child the MNP daily and the way to administer it properly.
2. In this household, who is favored during meal preparation or feeding?	The food prepared was solid. Kunu gyada was given to the baby boy and he ate separately

How is this favoritism expressed?	The baby ate in a separate bowl<br>The food was in solid state.
3. How could we promote the MNP supplement for this family that would attract their attention?<br><br>Who would they listen to?	MNP can be promoted to this family through the mother. It can also be promoted through the radio<br>Religious and community leaders are well respected so they will listen to them. The husband is the most influential in the household because he holds the final decision power in the house.
4. In this household, what would be the best ways to promote the MNP but limit how much it would be shared?<br><br>Why?	The child should be fed first with a portion of food that contains MNP before eating with the rest of the family.<br>Ensure the mother understands that MNP is meant for children from 6 months to 23 months<br>Ensure the older children are not present during MNP administration.

5. What other issues and concerns did you see with this family that are important for us to consider with regard to introduction of an MNP?<br><br>Why?	The only concern is the mother expressed how MNP has helped her son recovering from rashes on his body. So the fear is she might try to give the other children when they fall ill.
6. Based on your experience observing this child today, do you believe reactivity negatively impacted your ability to see the food-related behaviors of the child?<br><br>Please explain your answer in detail.	No. the child did not show any sign of reactivity during meal. The child ate without noticing the presence of a stranger. The mother did not forcefully feed him because he readily opened his mouth to eat. The baby finished the portion with the MNP and was given more kunu which he could not finish.
